# Sensing Cell-Culture Assays with Low-Cost Circuitry

**DOI:** 10.1038/s41598-018-27295-3

**Published:** 2018-06-11

**Authors:** Pablo Pérez, Gloria Huertas, Andrés Maldonado-Jacobi, María Martín, Juan A. Serrano, Alberto Olmo, Paula Daza, Alberto Yúfera

**Affiliations:** 1 Dto. Biología Celular, Facultad de Biología, Universidad de Sevilla, Av. Reina Mercedes no 6, 41012 Sevilla, Spain; 2 Instituto de Microelectrónica de Sevilla, IMSE, Universidad de Sevilla, Av. Américo Vespucio sn, 41092 Sevilla, Spain; 3 Dto. de Electrónica y Electromagnetismo, Facultad de Física, Universidad de Sevilla, Av. Reina Mercedes sn, 41012 Sevilla, Spain; 4 Dto. Tecnología Electrónica, Escuela Técnica Superior de Ingeniería Informática, Universidad de Sevilla, Av. Reina Mercedes sn, 41012 Sevilla, Spain

## Abstract

An alternative approach for cell-culture end-point protocols is proposed herein. This new technique is suitable for real-time remote sensing. It is based on Electrical Cell-substrate Impedance Spectroscopy (ECIS) and employs the Oscillation-Based Test (OBT) method. Simple and straightforward circuit blocks form the basis of the proposed measurement system. Oscillation parameters **–** frequency and amplitude **–** constitute the outcome, directly correlated with the culture status. A user can remotely track the evolution of cell cultures in real time over the complete experiment through a web tool continuously displaying the acquired data. Experiments carried out with commercial electrodes and a well-established cell line (AA8) are described, obtaining the cell number in real time from growth assays. The electrodes have been electrically characterized along the design flow in order to predict the system performance and the sensitivity curves. Curves for 1-week cell growth are reported. The obtained experimental results validate the proposed OBT for cell-culture characterization. Furthermore, the proposed electrode model provides a good approximation for the cell number and the time evolution of the studied cultures.

## Introduction

End-point cell-culture protocols have been, and are being, extensively employed in many assays for characterization of cell properties at biology labs. These assays allow observing numerous biological processes. Their final goal is typically to analyse the cell population in a dish or Petri plate as a measured response or consequence from a given external stimulus or biomedical treatment. These classical protocols require a large quantity of samples. They are expensive in terms of both material and human effort^1^. Alternatively, Electrical Cell-substrate Impedance Spectroscopy (ECIS)^2,3^ represents a mature method enabling real-time acquisition of biological parameters (number of cells, cell activity, motility and size) through the measurement of the cell-culture impedance^4–6^. It can be also applied for any kind of cell in relation with the environment^3,7,8^. ECIS has the advantage of being non-invasive. Unlike end-point protocols, it avoids the death of cells over time. ECIS is also relatively inexpensive since only one sample or Petri plate is required for a performance curve.

Two main aspects must be considered when it comes to implementing ECIS. First, in order to properly perform accurate bio-impedance measurements, adequate circuits must be selected according to the targeted measurement technique^9,10^. The accuracy of the obtained results will jointly depend on the efficiency and precision of this technique along with the fine performance of its circuit realization. Secondly, it is necessary to develop reliable electrical models for electrodes and cells. These models are meant to translate measurements into answers to the fundamental question: how many cells are in the culture^7,11,12^? Several cell-electrode electrical models have been reported in the literature. For instance, magnitude and phase impedance have been derived using a first-order RC model^2^. In turn, this model gives rise to another one based on three parameters: R_b_, the barrier resistance between cells; h, the cell-electrode distance; and r_cell_, the cell radius. As an alternative, Finite Element Simulations (FEM)^11,12^ can be executed for solving the electrical field across the whole structure. This method introduces a new parameter to the model, R_gap_, describing the gap or cell-electrode interface resistance. These two models extracted from the literature consider either the cell confluent phase^2^ or a fixed area covered by cells^11,12^. Both aforementioned points, i.e. suitable circuitry and proper modelling, are open research problems for biomedical engineering these days.

In this work, a system for real-time monitoring of cell culture assays from any internet-connected device (laptop, cellular phone, etc) is proposed. The underlying circuits are simple because they directly arise from the proposed bio-impedance technique. There are no strong specifications either for the Common-Mode Rejection Ratio (CMRR) in instrumentation amplifiers^13^ usually required for data acquisition, or for accurate AC voltage/current signal generators with programmable frequency for signal excitation^14,15^. The proposed circuitry measures the cell culture state by inserting it in a closed-loop oscillator. As a result, the frequency and amplitude of the quasi-sinusoidal output oscillations are a function of the cell number in the culture. The expected sensitivity curves for the system are theoretically obtained from the cell size and density, and the proposed electrode model.

The manuscript is structured as follows. Material and methods section describes the applied assay protocol. This section also includes the electrode-solution model (in our case, culture medium) useful for cell-electrode characterization as well as the procedure to develop meaningful cell-microelectrode models. The implemented circuit blocks are then described and their main functionalities, along with the design of the sensitivity curves derived for electrical measurement. Experiments carried out to model commercial electrodes, and their application to real-time cell culture monitoring assays, are presented in Experimental results section. Finally, Conclusions section summarizes our results, comparing them with the results obtained from the classical Petri plate based method for cell culture test.

## Material and Methods

### Cell line and culture conditions

The cell culture was carried out on a Chinese hamster ovary fibroblast cell line, AA8 (American Type Culture Collection). AA8 cells were cultured in McCoy’s medium supplemented with 10% (v/v) foetal calf serum, 2 mM L-glutamine, 50 μg/ml streptomycin, and 50 U/ml penicillin. Cells were routinely sub-cultured. The cell line was maintained at 37 °C in a humidified atmosphere with 5% CO_2_. They were always in exponential growth phase during the experiments.

### Electrodes

Commercial electrodes 8W10E PET, from Applied Biophysics (AB)^16^, were employed for cell culture assays (http://www.biophysics.com/). This multi-well is composed of eight separated wells, each one including ten circular 250- μm diameter bio-compatible gold microelectrodes.

### Cell growth

We conducted a basic growth assay. Cells were at in the incubator during one week. They were initially plated at a density of 2500, 5000 and 10000 cells/0.8 cm^2^ in multi-wells from AB. Cell growth was measured for seven days, with an observation period of 1 hour for each well time evolution from the beginning of the experiment. Petri-plate cultures were also conducted, featuring the same cell density for the sake of further comparison with the proposed bio-impedance test.

### Electrode-electrolyte model

**Figure 1:**
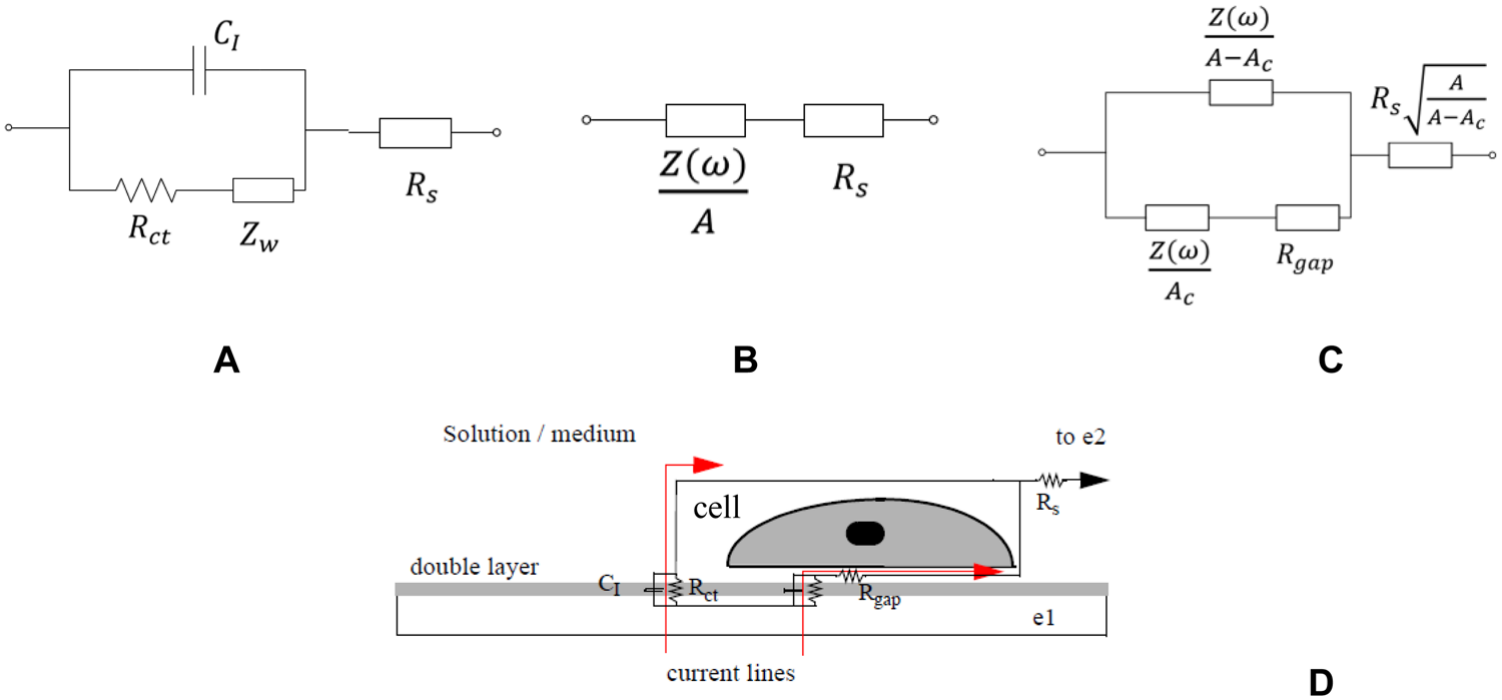
(**A**) Electrical model components of one electrode in contact with an ionic solution. (**B**) Simplified and area-normalized model in Fig. 1A without Warburg impedance. Z(ω) represents C_I_||R_ct_. (**C**) Proposed model for cell-electrode using a R_gap_ resistance – which models the current flowing in parallel through the interface between the electrode and cell, depending on the electrode-cell distance – and the fill factor parameter (ff = A_c_/A). A is the electrode sensing area whereas A_c_ is the electrode sensing area covered by the attached cells. (**D**) Illustration of the R_gap_ effect. The current flows from electrode e_1_ to e_2,_ as a response to an applied AC voltage.

The electrode impedance in ionic liquids has been widely studied in the literature^7,11^. The main components identifying the electrical performance of a metal electrode inside a solution are four, as illustrated in Fig. 1A: (1) C_I_, the double layer capacitance; (2) R_ct_, the transfer resistance, modelling the current flowing through the electrified interface; (3) Z_W_, the Warburg impedance, due to limited mass diffusion from electrode surface to solution. The electron transfer resistance R_ct_ is in series with the limited mass diffusion impedance Z_W_, which is only relevant at very low frequencies. Finally, (4) R_s_, the spreading resistance, that consider the current travelling across the bulk solution. These four elements depend on the technology, medium and geometry. A small AC voltage signal must be applied as an excitation to work in linear region^7^.

### Cell-electrode model

**Figure 2:**
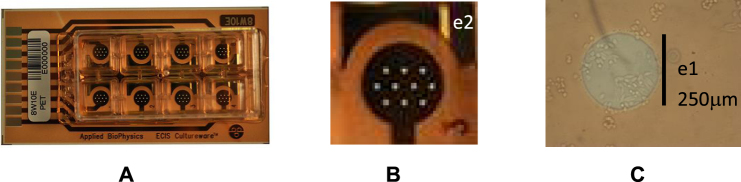
(**A**) 8W10E PET cultureware from AB^16^ with 8 wells of 0.8 cm^2^. (**B**) Cells are measured on top of the 10 circular gold electrodes, e_1_ (A_elec_), with total electrode area A = 10 × A_elec_. The sensing area is the sum of the 10 e_1_ gold electrodes, (**A**). (**C**) Photomicrograph of AA8 cells partially covering the area A_elec_ of a circular electrode.

Our practical setup for 8W10E PET culturewares is depicted in Fig. 2. A two-electrode impedance sensor is used: e_1_ is the sensing electrode comprising 10 parallel 250- μm diameter gold electrodes; e_2_ is the reference, usually ground-connected^2^. Since the area of e_2_ is much larger than that of e_1_, the cell location, number and size at e_1_ constitute the target to be detected (Fig. 2C).

The model in Fig. 1C implicitly assumes that the sensing area of e_1_ could be totally or partially filled by cells. For the two-electrode sensor shown in Fig. 2B, a 10e_1_ sensing area is defined by A, being Z(ω) the impedance per unit area of the empty electrode. i.e. with no cells on top. Considering partial coverage of the electrodes, let A_c_ denote the cell-covered surface on electrode e_1_. The impedance response associated to a non-covered or empty surface is defined by Z(ω)/(A − A_c_) whereas Z(ω)/A_c_ is the impedance of the covered area. The resistance R_gap_ considers the current flowing laterally through the electrode-cell interface. For an empty electrode, the impedance model Z(ω) corresponds to the circuit in Fig. 1B. The e_2_ electrode is normally large and connected to ground. Its impedance is small enough to be neglected. The parameter ff, called fill factor, equals zero for A_c_ = 0 – that is, for no cell coverage on e_1_ electrodes – and one for A_c_ = A – that is, for full cell coverage on e_1_ electrodes. Finally, Z_c_ (ff = 0) = Z(ω) is the magnitude of the electrode impedance with no cells. The fill factor is thus employed, together with the estimated cell size, to determine the area covered by the cells and the cell number.

### Implemented circuit

**Figure 3:**
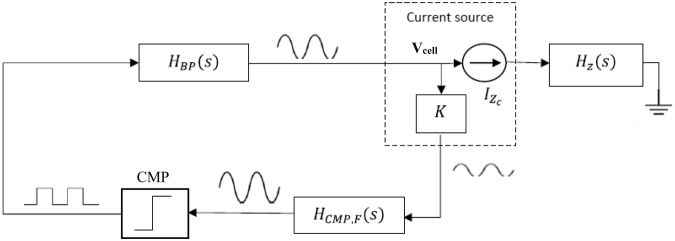
Simplified circuit block diagram proposed for measurement. It comprises the bio-impedance block H_z_(s), including Z_cell-electrode_, the comparator – K, H_CMP,F_(s) and CMP – and the band-pass filter H_BP_(s).

**Figure 4:**
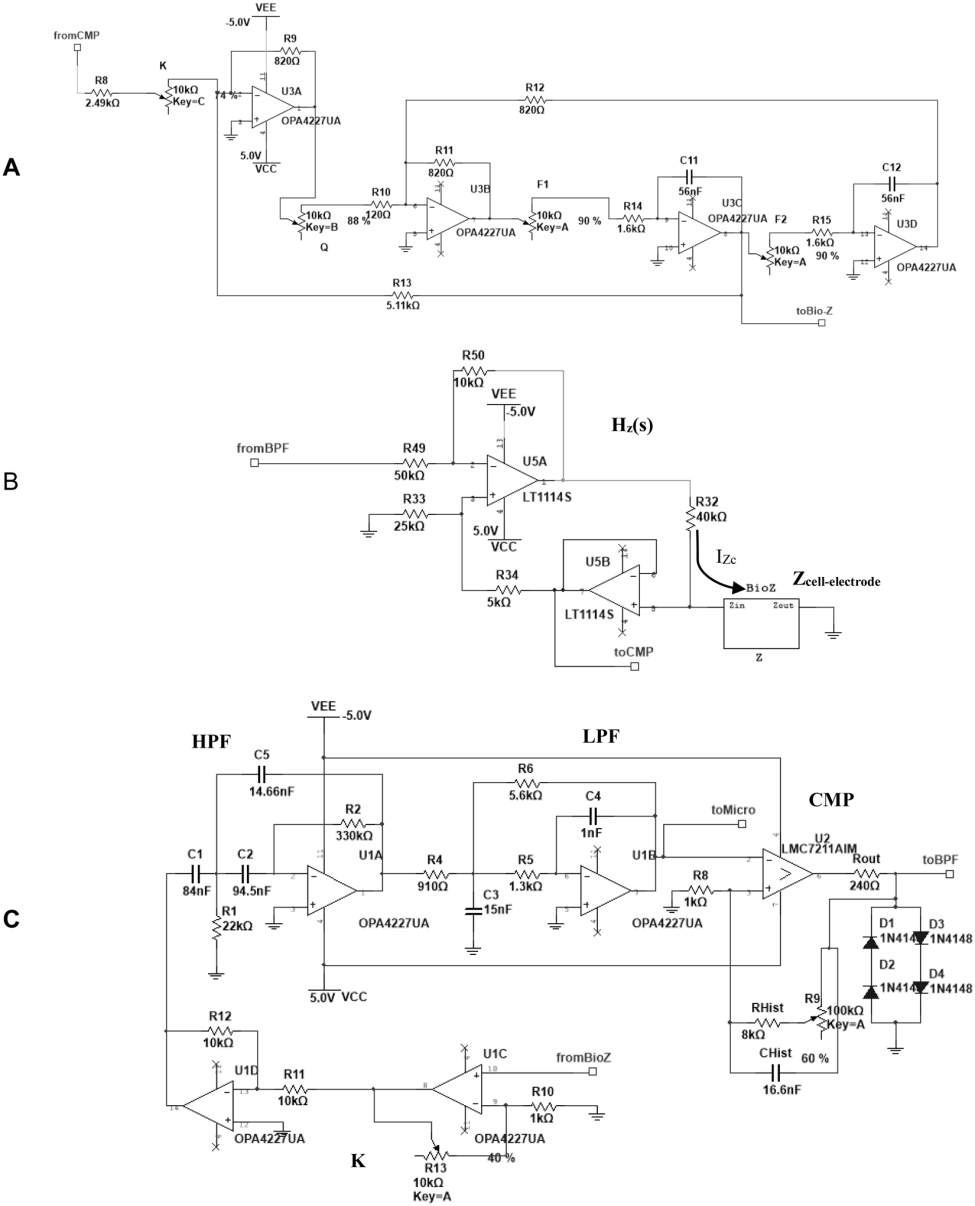
Circuits schematics employed for (**A**) Band-Pass Filter (BPF), (**B**) Bio-impedance block and (**C**) Comparator.

The proposed circuit for bio-impedance measurements avoids the use of high-performance^9,17,18^ circuitry or equipment, as well as the need for accurate current/voltage generators^15^, instrumentation amplifiers^13^ and precise demodulation circuits^10^. This is accomplished by turning the bio-impedance into a voltage oscillator whose oscillation parameters (f_osc_, a_osc_) are dependent on and proportional to the biological sample under test. A simplified circuit diagram is depicted in Fig. 3. Cell cultures are incorporated to circuit analysis through the electrode-cell impedance, Z_cell-electrode,_ introduced when cells are being cultured on ECIS electrodes. The Z_cell-electrode_ is included at the H_z_(s) block in Fig. 3. Circuit design is therefore driven not for a maximum normalized resistance value^8^, but for optimal circuit oscillation conditions^19^. This circuitry works as a voltage oscillator. It is characterized by two oscillation parameters: f_osc_ and a_osc_ at the output voltage signal V_cell_. The circuit obtains oscillation parameters correlated with the cell number, according to the electrode-cell model previously described, or similarly with the fill factor parameter. This process is monitored in real time using a remote sensing system^20^. The simplified block diagram is shown in Fig. 3 whereas the circuit schematics are depicted in Fig. 4. The building blocks for these circuits are operational amplifiers, resistances and capacitors.

The second-order Band-Pass Filter (BPF) allows the selection of the frequency at which the oscillator is tuned. The Q factor must be high enough to reduce the total harmonic distortion at the voltage oscillation signal (V_cell_) but also low enough to permit a wide frequency dynamic range. In this particular case, Q = 10. The BPF cut-off frequency, f_o_ = 1 kHz, was set according to the features of both the feedback loop and the bio-impedance sample to be sensed. The amplitude of the voltage V_cell_ supported by the culture is limited to preserve the linear response of the electrodes. Likewise, the current through the cell culture is limited to a maximum amplitude level of 20 µA. The BPF circuits are shown in Fig. 4A. The bio-impedance block is depicted in Fig. 4B. It is built upon a current source, I_Zc_, which is independent of the cell load thanks to a feedback path. Its function is to insert the electrode-cell impedance at the closed-loop transfer function. The output voltage, V_cell,_ is limited to 50 mV in order to attain linear response from the electrodes. To multiplex the eight electrode channels, a modified amplifier is employed to ensure that all of the electrodes – except for those ones being measured – have both terminals *e*_1_ and *e*_2_ connected to ground. The comparator circuit is shown in Fig. 4C. It incorporates a hysteresis window for input noise reduction that increases the loop stability in the oscillator response. Prior to the comparator, the low and high frequency components of the voltage signal V_cell_ are removed by a band pass filter – HPF in series with a LPF in H_CMP,F_(s). This signal is also amplified by a factor K = 100, simplifying the comparator operation. The data acquisition and wireless communication functionalities rely on various digital devices, including a digital section based on an ARM Cortex-M7 microcontroller device. This device has a rich set of peripherals. In particular, we exploit its Analog to Digital Converters (ADCs) for sampling data, and its Real-Time Clock (RTC) for synchronization. General-purpose input-output pins from the microcontroller can activate the analog signals and multiplex the wells to the sampled. Furthermore, ARM Cortex-M7 devices implement a Floating Point Unit (FPU) enabling them for on-chip execution of signal processing algorithms. A Bluetooth module was included in the system to facilitate wireless communication. The µP system inside the cell-culture incubator chamber is in standby mode most of the time. The system is battery powered with a model Ansmann 7.4 V Li, being the power consumption for a week assay of 985 mW. The variables for experiment control (sample time, for instance), can be defined and modified by the user via the web application. This interface also shows the data collected from the experiment in real time. The data plotted on the web application are the frequency and the amplitude obtained from the microelectrodes, i.e. the V_cell_ signal. The interface operates in real time and online. It can be checked on http://jarvis.dte.us.es/mixcell. None of the physical, chemical or biological factors of the experiments are affected by the wireless communication. Temperature, humidity and battery voltage level are continuously measured by the system.

### Impedance measurements with the HP 8591A Spectrum Analyser

A HP 8591A Spectrum Analyser^21^ was used to obtain the magnitude and phase of Bode plots for electrode characterization, both with medium only and including also cells. This characterization was required to select the correct operation frequency range for the proposed oscillator during its initial design stage.

## Experimental Results

### Electrode Model

**Figure 5:**
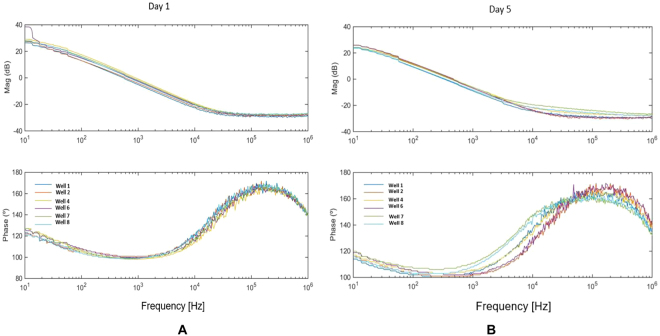
Impedance spectrum of one 8W10E PET electrode, both with medium and cells: magnitude and phase responses, measured with the HP-8591A at day 1 (**A**) and day 5 (**B**) of the experiment, for W1: 2500 cells, W2 and W6: medium. W4: 5000 cells, W7 and W8: 10000 cells.

The HP 8591 A Spectrum Analyser together with an inverting amplifier with 34.9-dB DC gain were employed to carry out a performance test aiming at measuring the three components of the electrical model of 8W10E PET, namely C_I_, R_ct_ and R_s_, in contact with medium. This test also rendered the impedance components of the electrodes in addition to the medium versus frequency characteristic – magnitude and phase. Pole-zero extraction from impedance Bode plots constitutes a first approach to extract electrode parameters. In Fig. 5A, a pole is located at around 8 Hz for wells 2 and 6 with medium, while a zero is located at 10 kHz, leading to R_ct_ = 618 k Ω, C_I_ = 32.2 nF and R_s_ = 495  Ω. These values are used for initial calculations when an electrode-medium electrical model is required, for example, during the design of the circuit in Fig. 3. However, two effects must be carefully considered in this process: first, the dispersion values from well to well; second, the time evolution of electrode parameters due to electrochemical activity on the electrode-to-medium interface along assays.

### Cell-Electrode Model

From the electrode-medium electrical model just defined, we can derive the corresponding cell-electrode electrical model based on three fundamental parameters: the fill factor (ff), the electrode area (A), and the resistance of the gap section (R_gap_) illustrated in Fig. 1C. A measurement similar to the one described in the previous section was carried out in an experiment where cells grew on top of ECIS electrodes. The magnitude and phase impedance responses per well were measured for five days with the HP 8591A Spectrum Analyser. The responses for day 1 and 5 are depicted in Fig. 5. These measurements prove that significant changes on magnitude and phase occur due to the increasing number of cells. Notably, phase changes match the targeted evolution for the selected band-pass filter peak frequency in the OBT feedback, thereby achieving good frequency sensitivities.

Following a similar process as the previous section, we obtain the electrode parameters C_I_, R_ct_, R_s_ and R_gap_, as well as matching values for R_gap_ in the range of (500  Ω, 1000  Ω). However, we must highlight that (1) for the medium wells, the electrode performance changes over time and, (2) between equal wells, their performance also changes due to mismatching effects, so the electrode parameters will be different from well to well and will also vary over time. Both effects make it difficult to find reliable parameter values for the electrical electrode model.

### Circuit expected performance: frequency and amplitude ranges

**Figure 6:**
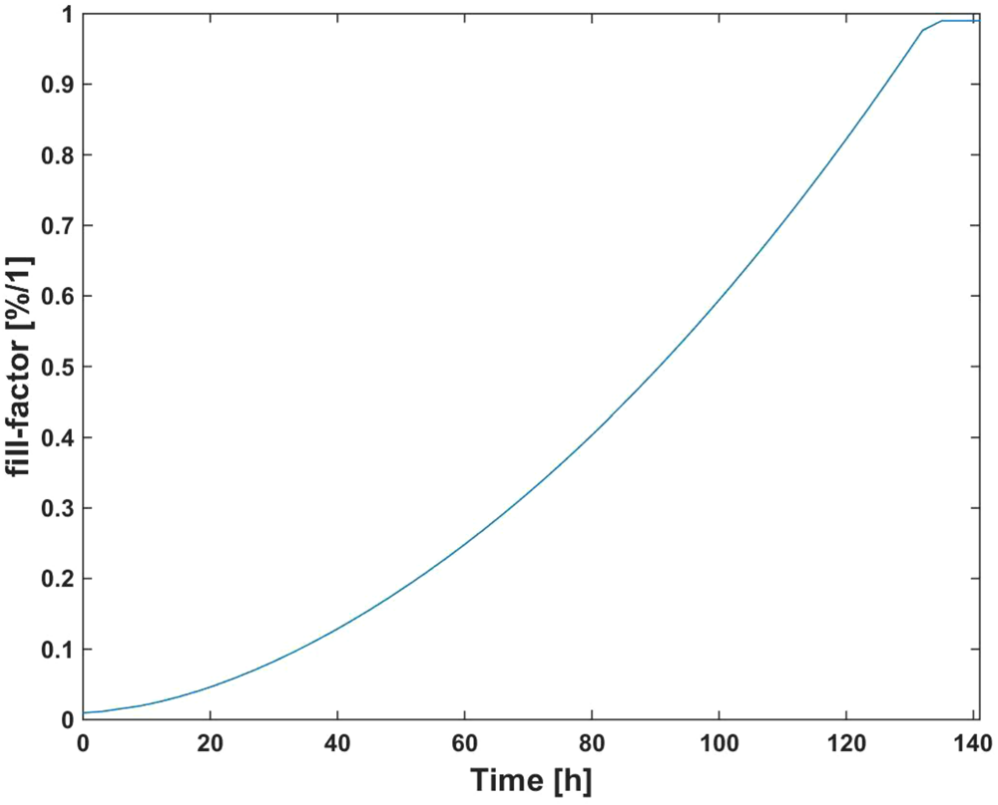
Expected values attained from Eq. (1) to estimate the fill factor vs. time for N_o_ = 5000 cells.

**Figure 7:**
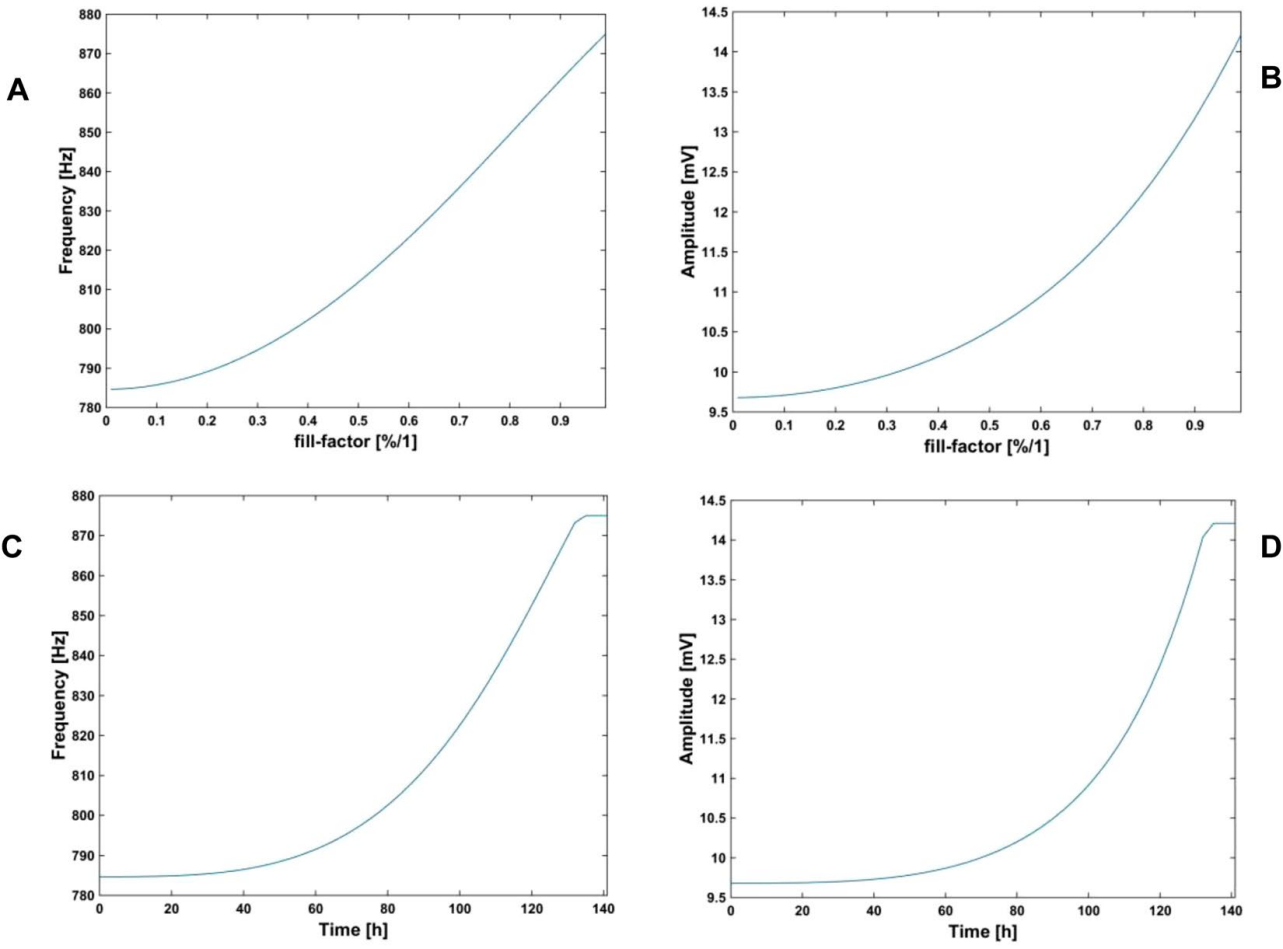
Frequency and amplitude values obtained from electrical simulations of the system in Fig. 3. The electrode parameters are experimentally extracted, whereas the ff prediction comes from Eq. (1). The values of the cell-electrode parameters are: R_ct_ = 618 k Ω, C_I_ = 32.2 nF, R_s_ = 495  Ω, and R_gap_ = 600  Ω. N_o_ = 5000 cells. (**A**) Frequency vs. fill factor. (**B**) Amplitude vs. fill factor. (**C**) Frequency vs. time. (**D**) Amplitude vs. time.

According to these experimental values for the electrode and cell electrical parameter models, we ran simulations to evaluate the expected frequency and amplitude ranges of the observed oscillation when cells were growing in a culture. These simulations also guided the design of the proposed circuits. Firstly, electrical simulations provided the frequency and amplitude response of the proposed OBT system when ff increases from 0 through 1 (cell growth). The circuit model shown in Fig. 1C was selected to emulate this scenario, setting ff = 0 for electrodes with no cells, and ff = 1 at confluence or monolayer state. Initial electrode parameter values were estimated from measured impedance responses in Fig. 5. They were then fitted considering the initial and final values (plateau phase) for frequency and amplitude measured at V_cell_ in the 5000-cell case. In order to have a model for the transient evolution of the frequency and amplitude responses in the system, we have estimated the time evolution of ff(t) from an exponential cell growth dependence:

1 $$ff(k)=\frac{{N}_{o}\,.{2}^{k}.{r}_{cell}^{2}.\pi }{{A}_{well}}$$

where N_o_ is the initial number of cells seeded at the well, k is the actual number of cell cycles (time in hours divided by the cell division cycle, in this case 18 h), r_cell_ is the cell radius (around 10 μm) and A_well_ is the 8W10E PET well area (0.8 cm^2^). Equation (1) renders an approximated value of the fill factor, plotted in Fig. 6 for an initial value of 5000 cells. Electrical simulations derived from this ff time dependence produce Fig. 7(A–D) for frequency and amplitude responses according to the electrode parameter values R_ct_, C_I_, R_s_ previously calculated, and R_gap_ = 600  Ω.

### Cell growth monitoring

**Figure 8:**
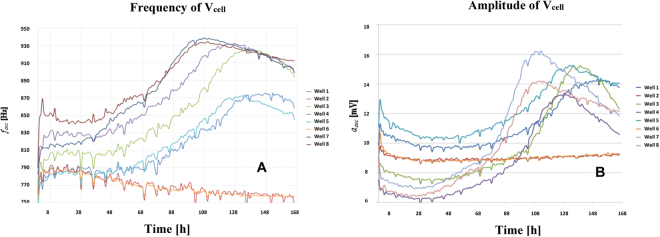
Measured time evolution of the oscillation frequency (**A**) and amplitude (**B**) of the voltage signal V_cell_. The curves correspond to 2500 cells (W1, W3), 5000 cells (W4, W5) and 10000 cells (W7, W8), seeded at t = 0 into separate well pairs. Wells W2 and W6 contain only medium. Dips in Fig. 8 are due to noise influence. Signals (currents and voltages) on electrodes and cells must be small enough to avoid damage in cells and preserve the linear model for the electrode-solution. These facts increase the sensitivity of measurements to noise sources and, decreases the Signal-to-Noise Ratio (SNR) in measurements.

**Figure 9:**
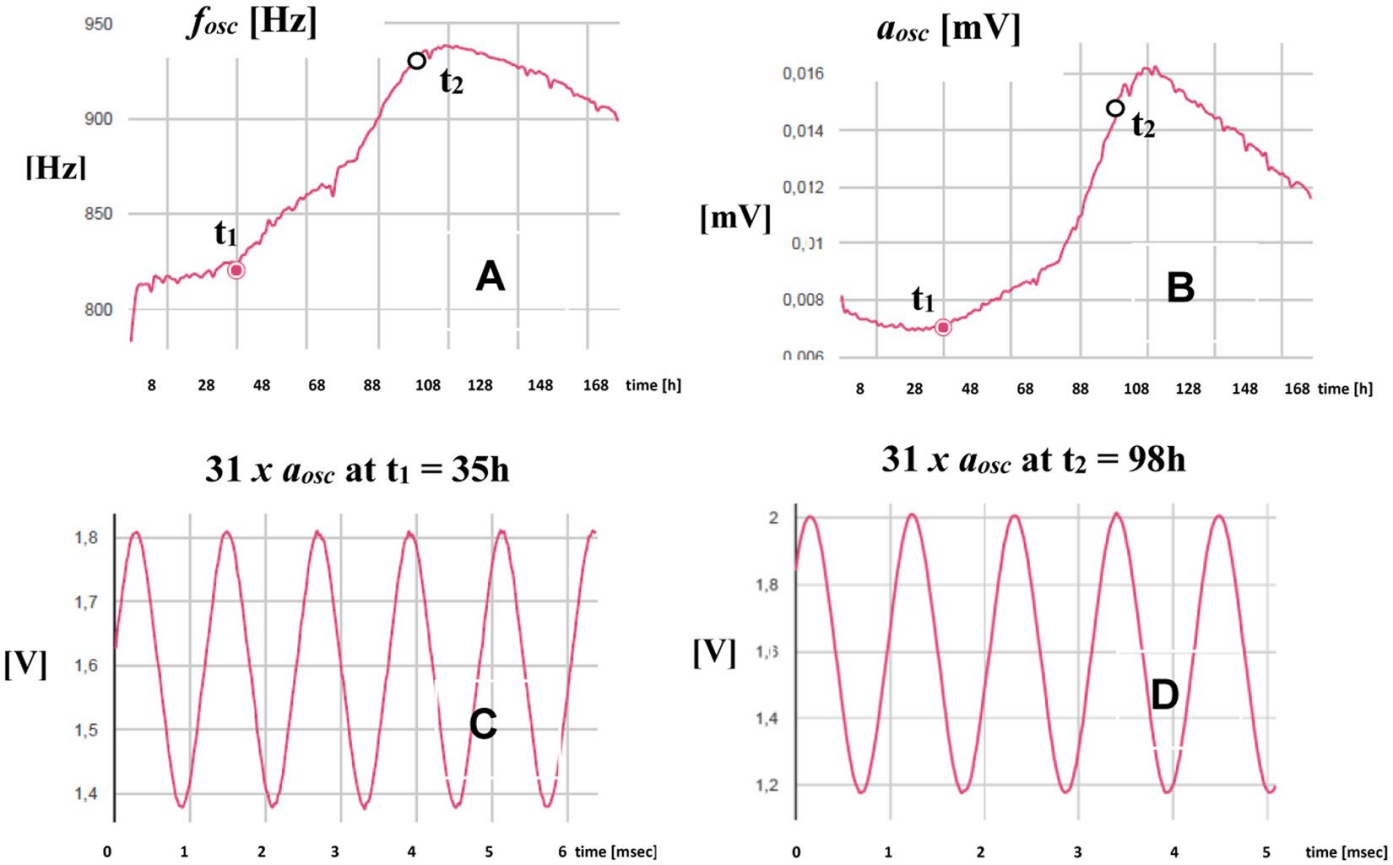
Time evolution of oscillation parameters at W7 extracted from the designed web page. Frequency (**A**) and amplitude (**B**) of the V_cell_ signal. Transient signals at t_1_ = 35 hours, f_osc_ = 824 Hz (**C**) and t_2_ = 98 hours, f_osc_ = 923 Hz (**D**). Note that a scaled factor of 31 is applied to the amplitude.

Cell growth assays were performed with AA8 cell line to validate the implemented measurement circuits. We also search fitting our model with the actual microelectrode-cell system and extract relevant biometric data, in this case, cell number or fill factor vs. time. In our setup, we initially seeded the medium with 2500 cells (W1, W3), 5000 cells (W4, W5) and 10000 cells (W7, W8) into separate well pairs. Two wells (W2 and W6) only contained culture medium. The eight wells were sequentially measured by introducing each well as the Z_cell-electrode_ impedance into the closed-loop feedback path in Fig. 3. Figure 8A shows the frequency evolution over time that we measured for seven days using 8W10E PET sensors with our circuit prototype^19^. Furthermore, the amplitudes measured at the output voltage, V_cell_, are plotted in Fig. 8B. The sample time was one hour, being this parameter completely tunable by the user. The frequency evolution proves that the frequency, effectively, increases over time as a consequence of increasing impedance caused by the growing number of cells attached to the electrodes, as it was expected from Fig. 7C. Initially, the cells require some time to adapt and recognize each well, so cell proliferation actually starts after around 24 hours, or even later. At the beginning of the experiment, there are no cells on the sensing electrodes in practical terms. The same frequency should therefore be measured in all of the wells, even at W2 and W6 where there was only medium. However, this initial value (called f_ini_) ranges from 770 Hz (W5) through 850 Hz (W8). This means that the electrode performance obtained from its electrical model could present dispersion values due to electrode mismatching. In addition, the Frequency Dynamic Range (FDR = f_max_ − f_min_) varies for each well: the maximum is for W7 whereas the minimum occurs for wells W1 and W5. This could also be caused by electrode mismatching. The frequency evolution for wells with medium (W2, W6) decreases over time. This could be interpreted as a dynamic evolution of electrical properties in electrode-medium interface owing to electrochemical reactions^7^. According to this result, the electrode model changes over time, so it is not quite correct to consider a “static value” for R_ct_, C_I_ and R_s_ components during the assay period. On the other hand, the amplitude evolution in Fig. 8B presents a similar behaviour, as depicted in Fig. 7D. It increases from an initial value, a_ini_, different from well to well, up to the confluence or plateau phase. The Amplitude Dynamic Range (ADR = a_max_ − a_min_) also varies among wells. Note that the amplitude values are very low (several millivolts). This is imposed to limit the maximum voltage and current amplitude through electrodes and cells, respectively. The amplitude at wells only with medium (W2 and W6) presents a slight increase over time. It seems to be less sensitive than the behaviour observed for the frequency. Transient signals at W7 are shown in Fig. 9 for t_1_ = 35 hours, and t_2_ = 98 hours. These signals are directly sampled by the uC ARM, and processed subsequently.

To compare frequency and amplitude evolution at different wells, we have normalized both responses defining the normalized frequency and amplitude as follows:

2 $${f}_{nor}=\frac{f(t)-{f}_{\min }}{{f}_{\max }-{f}_{\min }}$$

3 $${a}_{nor}=\frac{a(t)-{a}_{\min }}{{a}_{\max }-{a}_{\min }}$$

**Figure 10:**
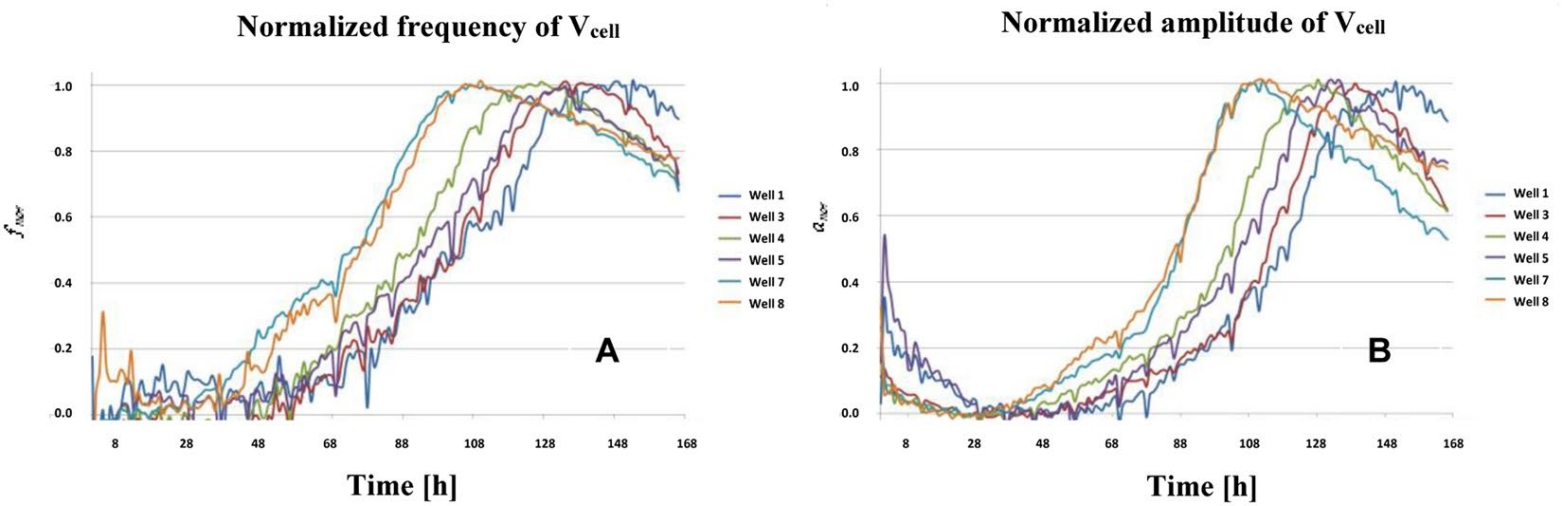
Normalized frequency (**A**) and amplitude (**B**) measured at V_cell_. The curves correspond to 2500 cells (W1, W3), 5000 cells (W4, W5) and 10000 cells (W7, W8), seeded at t = 0.

Figure 10A,B depict these normalized responses. Both frequency and amplitude evolutions show that well W7 and W8, seeded with 10000 cells, reached first the confluence state, while wells W1 and W3 reached this state the latest since they were seeded with 2500 cells. The time delay to search the plateau phase for wells seeded with 2500, 5000 and 10000 cells, is around 18–20 hours, i.e. around one division period of the cell line being tested. This demonstrates that our results are coherent with the expected performance. Considering a well area of 0.8 cm^2^, the frequency sensitivity at confluence phase is 100 Hz/0.8 × 10^8^  μm^2^ = 1.25 × 10^−6^ Hz/μm^2^. It means that for a circular cell of radius 10 μm, the sensitivity is approximately 4 × 10^−4^ Hz/cell = 0.4 mHz/cell. The sensitivity is calculated dividing the dynamic range of the frequency in Table 1 by the number of cells that can be fit in such well. A similar estimation for V_cell_ amplitudes renders a sensitivity of 0.03 μV/cell.

**Table 1:** Dynamic range experimentally observed for frequency and amplitude oscillatory responses.

Well	W1	W3	W4	W5	W7	W8
FDR [Hz]	94	114	101	93	123	93
ADR [mV]	4.60	7.65	7.00	4.85	9.19	7.60

## Discussion

Several aspects can be highlighted from these experimental results. First, the bio-impedance of the cell culture can be indirectly monitored in real time by measuring the frequency and amplitude from the proposed circuits. These signals are proportional to the cell culture bio-impedance, and hence to the number of cells. Second, the measurements performed for electrode-medium characterization shows a large dispersion from well to well when applying the electrode parameters proposed in our model. Moreover, there is a time dependence for frequency and amplitude responses in the electrode medium. This makes it impossible to initially fit the electrical model parameters of the electrode-solution and electrode-solution-cell.

We have carried out a study of cell growth evolution based on curves obtained from the circuit response. For this purpose, we applied the sensor models previously formulated. The frequency and amplitude of the oscillation were measured. The results show that cell growth can be characterized from these measurements. They increase monotonically as a direct consequence of the increasing number of cells up to the plateau phase. Their increment rate is proportional to the initial number of cells seeded in the culture. An experimental value of 0.4 mHz/cell has been estimated as sensitivity for the frequency response, and 0.03 μV/cell for the amplitude response.

**Figure 11:**
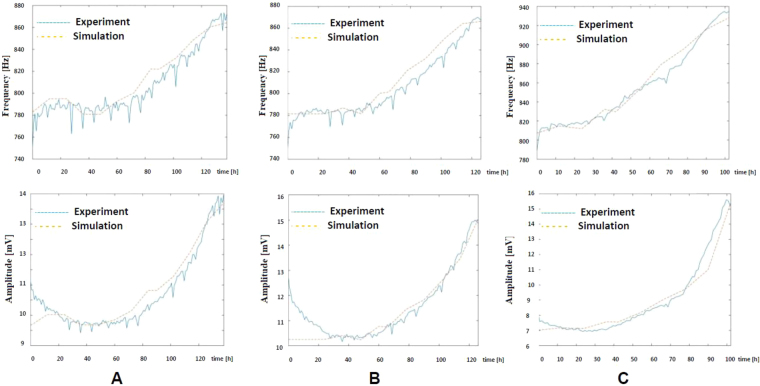
Simulated frequency and amplitude, with R_s_ in Eq. (4), measured in wells: (**A**) W1, 2500 cells, (**B**) W5 with 5000 cells and (**C**) W7 with 10000 cells. Electrical simulations include the models of cell-electrode and the circuits employed for measuring the cell cultures.

The expected dynamic range for frequency and amplitude derived from our electrical models does not exactly match the experimental results, as shown in Fig. 8. These deviations could stem from dispersion of the electrode parameters (C_I_, R_ct_, R_s_, and R_gap_). Note that the testing circuit is always the same, in contrast with the observed variation of the parameters in the electrode-solution electrical model over time. In this regard, an alternative fitting process for the R_s_ electrode parameter was applied over each individual well in order to accurately predict frequency and amplitude. In this process, we considered the initial and final measurements of frequency and amplitude, the cell size (10- μm radius) and an estimated cell division cycle of 18 hours, leading to,

4 $${R}_{s}={R}_{si}+{\rm{\Delta }}{R}_{s}.ff{(t)}^{n}$$

where R_si_ is the initial value of R_s_ calculated from the experimental amplitude at t = 0(ff = 0), and ΔR_s_ is its total increment, calculated at the end of the experiment for ff = 1. We set n = 4 for this approach. These hypotheses, together with the fill factor prediction given in Eq. (1) and the pole-zero based fitting process previously described, are integrated in the electrical simulations of the proposed circuits. The results are depicted in Fig. 11 in terms of frequency and amplitude estimations for the three initial numbers of seeded cells. The experimental measurements match well the prediction for the three cases, validating the estimation of the fill factor expected during the cell growing process.

**Figure 12:**
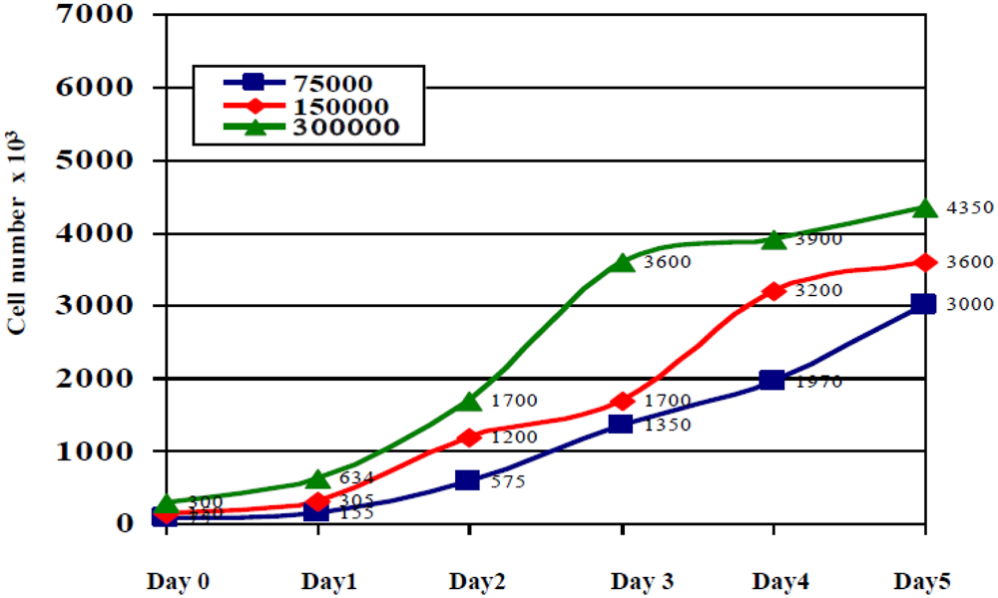
Measurements from AA8 cells; initially seeded cells: 75000 (blue), 150000 (red) and 300000 (green) in Petri-plates.

Finally, to check the number of cells on each experiment, we deployed cultures on Petri plates, seeding the same cell density. The growth curves obtained after five days are plotted in Fig. 12, for 75000, 150000 and 300000 initial cells. They correspond to 2500, 5000 and 10000 cell assays, with the same density at the wells, respectively.

**Figure 13:**
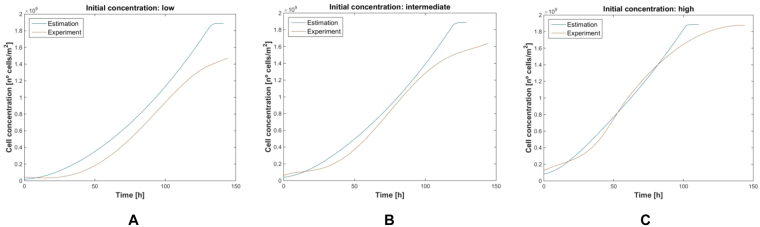
Evolution of the cell density measurement using Petri plates (brown), and estimated values resulting from frequencies and amplitudes measured in wells W1, W5 and W7 with the proposed circuits (blue), then decoded with the proposed electrical models. Cell densities were calculated for: (**A**) 75000 (2500) cells, (**B**) 150000 (5000) cells and (**C**) 300000 (10000) cells for each Petri plate (AB wells). Values employed in Eq. (1) are r_cell_ = 12.56 μm, and 20 hours for time division.

The proposed electrical model for the cell-electrode-medium system was applied to data in Fig. 12, achieving the best match for a cell division time of 20 hours and a cell radius of 12.58 μm. This period is higher than the expected value of time division (normally around 12–14 hours). Notice that the speed of the cell growth is slower at the start and at the end of the cell growth intervals. However, the proposed model in Eq. (1) always assumes an exponential dependence between the cell number and time. When using this model as “key” to decode our results, the experimental cell density is that in blue in Fig. 8 for 2500, 5000 and 10000 cells in wells W1, W5 and W7 respectively. These plots can be compared with that in brown in Fig. 13 derived from the same cell density data measured using Petri plates. The best cell density prediction is attained for high-density cultures, Fig. 13C, while for low density, Fig. 13A, the error is around 15%. A better cell density match could be obtained by obtaining a fine-grain evolution of the cell growth process over time.

## Conclusions

This work describes the application of low-cost circuitry for real-time monitoring of cell-culture assays. We present an approach for measurement developed upon oscillation-based test techniques and simple and easy-to-implement circuits. We have designed, fabricated and successfully tested these circuits performing the proposed techniques.

Commercial electrodes have been modelled for ECIS assays. These electrical models are required to decode the measurements since the electrodes are placed in the signal path, thus contributing to the sensor response. The electrode parameters exhibit a large dispersion of values, precluding any attempts to assign them a constant value. Therefore, we have also described a method to fit the value of each parameter and a modified expression for the evolution of the spreading resistance as a function of either the fill factor or the number of cells in the culture.

We have conducted assays for AA8 cell line and for several cell densities: 2500 cells/0.8 cm^2^, 5000 cells/0.8 cm^2^ and 10000 cells/0.8 cm^2^. The assays were measured and monitored with the proposed circuits for a week, demonstrating the correct operation not only of the circuits themselves but also of the battery-power system, wireless communication and web page monitoring.

We have compared our results with standard techniques of cell culture count, obtaining a maximum error of 15% in the cell number for low densities. This work assumes an exponential dependence for cell growth evolution. This dependence is employed to search and fit the values of the electrical cell-electrode parameters. By applying a more accurate time evolution model for cell growth, slower at the beginning and at the end of the cell culture period, cell count should be improved in future works.
